# Proceedings of the Medical Convention at Macon

**Published:** 1871-07

**Authors:** S. B. Hawkins, W. Duncan

**Affiliations:** President; Secretary


### PART III.

### Correspondence, Hygenic and Miscellaneous.

### PROCEEDINGS OF THE MEDICAL CONVENTION HELD IN THE CITY OF MACON, GA., JULY 5, 1871.

CITY HALL, MACON, GA., JULY 5, 1871.

Pursuant to an advertised call, signed by one hundred and fourteen physicians from various parts of the State of Georgia, the meeting assembled at 10 a. m., at the City Hall, Macon, Ga., and was opened by prayer by the Rev. Mr. Vaughn, after which Dr. Wm. R. Burgess, of Macon, in a few appropriate remarks, extended to the members present a cordial welcome on behalf of the Medical Association of Macon, concluding which, he moved that for the purpose of temporary organization, Dr. E. F. Knott, of Griffin, be requested to take the Chair, which was seconded and carried. On further motion, Dr. Wm. Duncan, of Savannah, was requested to act as Secretary. On motion of Dr. R. D. Arnold, of Savannah, it was

*Resolved*, That the physicians present proceed to register their names, together with the college at which they graduated, and the date of the same. The following list was then proposed by the Secretary.*

Dr. R. D. Arnold, of Savannah, then moved that a Committee of Credentials, consisting of three members, be appointed by the Chair, which being seconded and carried, the Chair appointed Dr. R. D. Arnold, of Savannah, Dr. S. B. Hawkins, of Americus, Dr. F. Walker, of Monticello. The registered list of members present was then handed to the committee, who retired, and after a few minutes consultation, returned and reported that all the names registered were from regular institutions in the United States.

Dr. Wm. F. Holt, of Macon, moved that a committee of five be appointed to nominate officers for the permanent organization of the Convention, which being seconded and carried, the Chairman

*This list is omitted for want of space.—[EDITORS.]

appointed Dr. Wm. F. Holt, of Macon, Dr. J. B. Hinkle, of Americus, Dr. W. T. Goldsmith, of Atlanta, Dr. John D. Fish, of Savannah, Dr. E. D. Alfriend, of Sparta.

The Committee presented the following names for the permanent organization of the Convention:

Dr. S. B. Hawkins, of Americus, President.

Dr. C. H. Hall, of Macon, 1st Vice President.

Dr. F. Walker, of Monticello, 2d Vice President.

Dr. W. Duncan, of Savannah, Secretary.

A motion was then made that the report of the Committee on Nominations be accepted as a whole, which being seconded and carried, the Chair announced the gentlemen nominated to be unanimously elected the permanent officers of the Convention. Dr. J. B. Hinkle, of Americus, then moved that a Committee of five be appointed to draft resolutions and report business for the action of the Convention, which being seconded and carried, the President appointed the following gentlemen: Dr. R. D. Arnold, of Savannah; Dr. C. B. Nottingham, of Macon; Dr. J. B. Hinkle, of Amerricus; Dr. G. G. Crawford, of Atlanta; Dr. W. A. Green, of Americus.

At this stage of the proceedings a number of gentlemen arrived, when Dr. P. H. Wright, of Macon, asked that those gentlemen be requested to present their names to the Committee on Credentials, in compliance with the resolution in reference to registration—Dr. S. B. Hawkins having been elected President of the Convention, appointed Dr. H. Wright, of Macon, to act in his stead, on the Committee on Credentials.

The registration of names having been concluded, the following list was presented to the Committee:

[This list is omitted for the want of space.—EDITORS.]

The Committee on Credentials, returned the following report:

The Committee respectfully report that the Physicians registered, all reported as graduates. Some are graduates of the Atlanta Medical College, since the disqualifying resolution of 1868. This resolution was rescinded at the last meeting of the Georgia Medical Association at Americus. While this Convention was called almost unanimously by Physicians opposed to the rescinding, yet it is bound by this rescinding until it shall be revoked at the next meeting of the Association, at Columbus, and to its judgment, we leave the merits of the case.

R. D. ARNOLD,

P. H. WRIGHT,

F. WALKER.

} *Committee on Credentials.*

A motion was made by Dr. E. D. Alfriend, of Sparta, that the report of the Committee be received, with the exception of that portion referring to the graduates of the Atlanta Medical College, since the disqualifying act of 1868, which being seconded and put to the vote, was decided by the President, affirmatively. Dr. Harriss, of Savannah, appealed from the decision of the Chair, and asked that the ayes and nays be taken. At this point, Dr. W. A. Love arose and asked the Committee to withdraw that portion of their report referring to the Atlanta Medical College; the report was then recommitted to the Committee, and all that portion referring to the Atlanta Medical College was withdrawn by them. A motion for adjournment was then made and lost.

The Committee appointed to draft resolutions and report business for the meeting presented the following report:

WHEREAS, The Legislature of the State of Georgia did, in the year 1858, make the following amendment to the charter of the Atlanta Medical College, viz:

"The Professors and Trustees shall constitute a board, who are hereby authorized and empowered to confer the degree of Doctor of Medicine upon such applicants, and under such circumstances as may, to the Professors, seem fit and proper; and the said Professors shall have power to change the chair of, remove, or fill the vacancy by the resignation, or otherwise, of any and every member of their Faculty."

*And whereas*, This unusual grant of power had remained unknown to the majority of the medical profession in the State of Georgia until the year 1868, and it was properly brought to the notice of the Georgia Medical Association by Dr. G. G. Crawford, at its annual meeting at Augusta, at which time and place the Association adopted the following resolution, presented by Dr. Sam. White:

"*Whereas*, The above amended charter confers unusual and extraordinary powers upon the Faculty of the Atlanta Medical College, whereby they are authorized to confer the degree of M. D. on persons, regardless of time or condition, save as to said Faculty may seem fit and proper, therefore

"*Resolved*, That we cannot recognize the graduates of said College that may hereafter receive their diplomas under the amended charter aforesaid."

"This preamble and resolution were adopted as a substitute for the preamble and resolutions of Dr. Crawford."

The said amendment to the aforesaid charter did, in the opinion of this Convention, confer not only unusual and extraordinary but improper powers dangerous to the cause of medical education, and the Georgia Medical Association was acting in the line of its duty as custodian of the honor and dignity of the profession within the State, in calling attention to it and making efforts for its repeal.

*And whereas*, While a petition for the repeal of this obnoxious amendment was pending before the Legislature of Georgia in 1868, the Atlanta Medical College presented a memorial containing the following statement:

"That the action of this Association which repudiated the Atlanta Medical College as a regular institution, had in it 'an utter absence of all the elements of truth,' and that the annual session of this Association, in 1868, was a meeting of physicians assuming to represent the medical profession of the State, and that such annual meeting was made up, almost entirely, of the Dr. Powell clique, of the city of Atlanta, and of members of rival schools, and that the whole affair (Association meeting) was gotten up and consummated for the very purpose of injuring the Atlanta Medical College. That the voice of the State Medical Society and of the profession was not heard. That the meetings of this Association are seldom attended by any but resident physicians, and a few others who have some interest to advance. That the annual session in Augusta, as evinced by the names and votes, was such a meeting of persons with interest to advance."

*Now be it Resolved*, By the Convention which is held in pursuance of the following call, signed by 114 physicians of the State of Georgia:

"From an interchange of views and opinions with the oldest Medical Societies and a large number of the most prominent physicians in the State, it is believed that it is desirable that a Convention of the Regular Physicians of Georgia should be held at some suitable time and place, to express the views of the Profession at large, as to the action of the meeting of the Georgia Medical Association, in reversing the judgment passed at three anterior consecutive meetings in 1868, 1869 and 1870, in relation to the Atlanta Medical College; and to elicit such an authoritative expression of opinion, as would serve as a guide to the next meeting of the Georgia Medical Association, to be held in Columbus, Ga., whereby all questions pertaining to the Atlanta Medical College shall be definitely and finally settled."

"The undersigned, therefore, hereby respectfully name Macon, Georgia, as the place, and the first Wednesday in July next, 5th, as the time for holding such Convention, and invite the presence and co-operation of all the regular Physicians of the State who are desirous of upholding the dignity and honor of the Medical Profession."

*Resolved, by the Convention*, 1. That the amendment to the charter on which the action of the Association was based, was a fixed fact, the record of it being spread on the Statute Book of the State, and that to say an action based on it, "had in it an utter absence of all the elements of truth," And it did not involve in it a reprehensible accusation of falsehood against a body of Physicians who really did constitute the only State Society in our borders, and were therefore in the legitimate exercise of their rights and duties in endeavoring to correct an abuse in the degree-conferring power.

*Whereas*, This subject matter was brought up at the meeting held at Savannah, in 1869, and an apology as follows was tendered:

'Dr. Orme then read the following communication from the old Faculty of the Atlanta Medical College:

'ATLANTA, GA., April 10, 1869.

'At a meeting of the Faculty of the Atlanta Medical College of 1868, held in the city of Atlanta March 16, 1869, the following resolution was unanimously adopted, viz:

*Resolved*, That this Faculty disavow any purpose to reflect upon the Medical Association of Georgia, either in the 'memorial' presented to the Legislature of 1868, or upon any other occasion, and that our Representatives, who may attend the meeting of said Association to be held in the city of Savannah on the 14th instant be, and they are hereby instructed to present this disavowal, together with that contained in said memorial. By order of the Faculty.

JESSE BORING, M. D.,
Dean of the Faculty.

WM. S. ARMSTRONG, M. D. Secretary.

On which the following resolution, among others, was passed:

*Resolved*, That a proper self-respect on the part of this Associatien, requires of the former Faculty of the Atlanta Medical College a distinct and unequivocal withdrawal of the objectionable language used in their recent memorial, and such withdrawal must be through the public journals of the State."

Which was refused, and in the afternoon of the same day, Dr. Charters offered the following preamble and resolution:

*Whereas*, The irregularity of which this Association complained at its last session, in the charter and management of the Atlanta Medical College, has been removed by the action of the last Legislature, and said College is now conducted upon principles which this Association approve; be it

*Resolved*, That the resolution adopted by the Association at its last session, discrediting the diplomas of the Atlanta Medical College, shall have no reference to diplomas that may be conferred by that institution hereafter."

Dr. Harriss moved to amend the resolution by adding the following:

"Provided the present Faculty, under the new charter, will repudiate the action of the Faculty existing at the time of the passage of the resolution, during the meeting at Augusta."

This amendment was adopted, when the preamble and resolution, were on motion, adopted.

*And Whereas*, When the meeting of the Association took place at Macon, April, 1870, a communication from the Trustees and Faculty of the Atlanta Medical College was read, and on motion, was laid on the table and the following resolution adopted:

*Whereas*, The late Faculty of the Atlanta Medical College, having failed to comply with the requirements of the "Georgia Medical Association" at Savannah;

*Be it Resolved*, That their names be stricken from the roll of membership of this Association, with the exception of Dr. A. Means."

*Resolved*, That in the opinion of this Convention the three meetings of the Association, held regularly at Augusta, Savannah and Macon, were regular meetings of the regularly organized State Society, and any imputation on the honor and integrity of the members who attended these meetings is an unprovoked insult on them, and on the profession at large in the State.

*And Whereas*, At the recent meeting at Americus, April, 1871, while the former charge of mere personal motives brought by the Atlanta Medical College against the Association remained unretracted, the meeting passed the following:

*Whereas*, The controversy between the Faculty of the Atlanta Medical College, the Atlanta Academy of Medicine, the Fulton County Medical Society, or members of the same who are members of this body, or between said bodies or individuals and any other members of this Association, are of a personal character, and ought never to have been introduced into this body.

"*Therefore Resolved*, That all action of this Association upon these controversies be rescinded, and be no longer regarded as a part of the archives of this body."

Thereby reiterating the offensive and insulting charge of "personal" motives.

*Resolved by this Convention*, That the three meetings of the Association of 1868, 1869 and 1870, were regular meetings of the only State Society existing in our midst; that their actions were in due accordance with parliamentary law and their unquestionable rights, and that this Convention hereby approves of them, and that with this endorsement and approval of this Convention, respectfully recommends to the next meeting of the Association to reverse and rescind the action of the last meeting in relation to the preceding meetings.

*Resolved*, That this Convention will not entertain any matter pertaining to any disputes outside of the Georgia Medical Association; and in their action at present they claim to act with a sole view to the preservation of the peace, dignity and honor of the medical profession in the State of Georgia.

A motion to adjourn until 3 o'clock, was then made and lost. A further motion to adjourn until 2 1-2 p. m. was made and carried. The meeting then adjourned.

AFTERNOON SESSION.

The meeting was called to order by Dr. S. B. Hawkins, President. On motion of Dr. W. A. Greene, of Americus, it was

*Resolved*, That an assessment of two dollars be made upon each member of the Convention, for the purpose of defraying the expenses of the same.

On further motion it was

*Resolved*, That a Treasurer be appointed, whereupon the Chairman appointed Dr. W. A. Greene, of Americus.

The minutes of the morning session were then read, when quite a discussion arose as to the propriety of the Secretary's recording that portion of the report of the Committee on Credentials, referring to the registering of graduates of the Atlanta Medical College after the disqualifying act of 1868.

It being decided that it was the duty of the Secretary to record the transactions of the meeting fully, and in the order of their occurrence, the minutes of the morning session were then confirmed.

Dr. Robert Battey, of Rome, after some remarks in relation to the unfortunate controversy between the Georgia Medical Association and the Atlanta Medical College, moved that a Committee of five be appointed by the President to confer together and report such plan as might be deemed most advisable, whereby all questions pertaining to this difficulty, might be satisfactorily adjusted.

Dr. Ridley, of LaGrange, made a few remarks, stating that he had attended this meeting in the interests of peace, that he recognized that all the members of the Profession desired to see this whole difficulty settled, but that the only way that it could be settled, in his opinion, was by "compromise." Concluding his remarks, he moved the "previous question." Several members, in vain, attempted a hearing, and in the confusion which resulted, Dr. Green, of Americus, moved an adjournment until 10 o'clock, A. M., the next day. Dr E. S. Kerksey, of Columbus, offered, as an amendment, that the meeting adjourn until two days after the next meeting of the Association, in Columbus, Ga.—the vote being taken, the Presiecent decided the motion lost, the yeas and nays were then called for, and resulted 39 in favor of adjournment, to 38 against adjournment. The meeting then adjourned to meet two days after the next meeting of the Georgia Medical Association, at Columbus, Ga.

S. B. HAWKINS, M. D., *President.*

W. DUNCAN, M. D., *Secretary.*

